# Abstracts and Gleanings

**Published:** 1886-05-20

**Authors:** 


### ABSTRACTS AND GLEANINGS.

**Retention of Urine from an Unusual Cause.**—Dr. J. Halliday Croom, M. D., in *Edinburgh Medical Journal*, February, 1886: A gentleman brought his wife to my consulting room, telling me that she was in great distress from a swelling in her belly. I found on examination a tense round dull tumor extending up to the umbilicus. She informed me that she had passed water recently, and had a constant desire to do so. Before proceeding further, I did what I always do in all pelvi-abdominal tumors, viz., introduced a catheter. I removed about two quarts of urine. Being at a loss to know the cause, on inquiry, found that she had been married two days previously, and that since the night of her marriage she had complained of more or less distress, and had passed water only in very small quantities since. On examination per vaginam, I found that the hymen, which was unusually thick and fleshy, and which was of the usual crescentic form, had been completely torn in the centre, and that the mucous membrane covering the posterior vaginal wall had been deeply lacerated for at least an inch. At the time of first intercourse there had been considerable pain, and some hemorrhage, the patient stated that afterwards she had felt sick and faint. Both parties believing what had occurred to be the usual state of matters, the husband renewed his attempts later on in the morning, but since that time no further intercourse had taken place. Twice before I have seen as a result of violent intercourse considerable and even deeper laceration of the posterior vaginal wall than in the present case, but unassociated with retention of urine. Secondly, the case belongs to that class of retention cases which are reflex in their causation. It belongs exactly to the same class as retention of urine in the puerperal woman from laceration of the perineum, or as in retention arising from urethral caruncle. The retention of urine is, in my opinion, not due, as some have said, to the patient, voluntarily retaining her urine from the dread of allowing it to come in contact with a raw tender surface, and as a consequence, the retention becomes involuntary from over distention and temporary paralysis of the muscular coat of the bladder. Rather, it is due to a reflex mechanism. It seems to me that this case, in common with all those of perineal laceration accompanied with retention, arises from tonic spasm of the sphincter vesicæ, caused by the stream of afferent stimuli reaching the centre from the nerve-endings in the lacerated wound.

**Paraldehyde.**—Compared with chloral, paraldehyde (says the American Journal of Medical Sciences) has these advantages:

"It is less irritant, and better supported by the stomach. It is not a heart poison. It is a more efficient antidote to strychnine. But it has less analgesic action, and less power to relieve pain than chloral, and hence when insomnia is caused by pain the latter is preferable, and morphine is still more efficient. In nervous insomnia, in that due to the abuse of alcoholic drinks, paraldehyde is much superior to chloral. Especially is paraldehyde most useful in the different forms of mental disorder. They have also shown that it is a valuable hypnotic in certain cases of insomnia with the excitement occurring in the course of some cerebral affections, in the convulsive neuroses, and especially in epileptic crises, and in the multiform manifestations of hysteria. Dr. Dujardin-Beaumetz has also treated many cases of morphiomania by paraldehyde, giving 45 to 60 minims a day. It does not appear to lose its effect by repetition, since the same results have been obtained through months of treatment.

"Mr. G. F. Hodgson has had experience with paraldehyde which supplements the observations of Dr. Dujardin-Beaumetz. Mr. Hodgson finds it to be a hypnotic of great value, in that it produces sleep like the natural state, promptly and without any unpleasant after effects. He regards it as the most appropriate hypnotic in the insomnia of gout, in mania,, hypochondriasis, delirium tremens, migraine, and in the wakefulness of ordinary diseases. His prescription is as follows:

"℞. Paraldehyde.............................ʒ i,

Spiritus chloroformi.......................*m* xv,

Pulv. tragacanth, comp.......................℈ i,

Syrup aurantii.............................ʒ iv,

Aquam.............................q. s. ℥ iij. M.

"This dose is sufficient in the milder cases, but must be repeated in the more severe."

**Amenorrhœa.**—Dr. W. H. Tate, Greensburgh, Ohio, in Peoria Medical Monthly, says: Having read an article in the August number of the Monthly on manganese in the treatment of amenorrhœa, by Dr. A. R. Hicks, it reminded me of several cases in my hands treated with potassæ permanganas, one or two of which I will refer to.

About the first of March, 1885, Miss H., a girl about 18 years of age, called at my office for treatment, her menses having been absent for several months, from the effects of getting her feet wet which resulted in suppression of the monthly flow.

Although she had been remarkably healthy previously, and had menstruated regularly, yet at the time she presented herself for treatment there were evident symptoms of decline, viz., nervous chills, a cough characteristic to such ailments, night sweats, emaciation, etc. I described the following:

℞. Potassæ permang.............................1 dr,
Aqua pura.............................8 ozs.

Dissolve.

Sig. One teaspoonful every four hours, commencing a week or ten days before the expected return of the catamenia.

If necessary this amount may be continued for two or three weeks if it does not interfere with the functions of the stomach.

About the latter part of August last Mrs. B., a married woman about 20 years of age, also stated that she had not experienced her menstruation for about three months and applied for treatment. I resorted to the permanganate which was continued for a week or ten days with the most happy result, producing a most copious discharge of the menstrual fluid.

I have since treated quite a number of cases suffering from suppression of the menses and have found the remedy above alluded to as efficient and reliable as any other that I have ever resorted to for such purposes. All the cases alluded to yielded very promptly to the drug, which brought about results far more pleasing than I had anticipated.

**Veratrum in Typhoid Fever.**—Almost every practicing physician knows the great and never failing effects of the fl. ex. verat. vir. in all acute inflammations, high temperature; also a great many physicians prize it highly in the treatment of various forms of erysipelas, etc.

But I wish to speak of its effects in typhoid fever with a high temperature, a dry skin, sordes on teeth and a dry parched tongue, with pulse frequent and feeble. (Now I am aware that some one will say that something else would have succeeded better, and that the plan of treatment I shall here lay down, is not simon-pure Eclecticism; but I care not what is said if my treatment succeeds; it is when the treatment fails that criticism hurts.) In seven cases treated, I used the fl. ext veratrum vir. in sufficiently large doses to hold the temperature down to 103 degrees, beginning its use as the fever reached that point, and continued its use until the temperature in the morning came down to 98½ degrees, let that be two, three or even five weeks, giving it as follows:

℞. Fl. ext. veratr. vir............................fl. ʒ 3,

Simple syr. squill............................fl. ʒ 6.

M. Sig. Begin with nine drops every three hours, and increase one drop every dose until the fever is controlled and held below 104 degrees.

I have administered as high as 21 and 22 drops every three hours for a whole day and night. As soon as the least moisture appears on the skin, or the temperature starts down, I decrease the dose of veratrum at the rate of three drops at a dose. Of course I have a thermometer at the house, and have the temperature taken before each dose while giving the large doses.

Alternated with the above, I always give five to seven drops of turpentine (the oil) in mucilage of acacia every three hours. Also, a flannel cloth wrung out of a mixture of spirits turpentine and mutton lard, equal parts, is kept constantly on the bowels until the skin becomes reddened. Then this is left off a few days, and reapplied if tympanitis continues.

*Nourishment.*—Sweet milk, alone, generally is given just before or just after the turpentine emulsion, every three hours, with as much regularity as the veratrum, and as much as the patient will take, too—the more the better.

Never let a typhoid patient remain in one position too long, but have him turned—not turn himself—from one side to the other every three hours. This last refers only to patients who linger, and by lying too long on one side or the back causes conjestion of the lungs.

In a practice of nearly five years in this county (Franklin), I have treated twenty-three cases of typhoid fever, with a loss of one case—treated with quinine—and the plan above indicated is the one that has given the best results. Fever usually begins to decline about the fifteenth, or anyhow the twenty-first, day. In the above plan I have never been troubled with hemorrhage, strangury—as in cases where blisters were used—and very little trouble has arisen from diarrhœa. Patients sleep well generally, and make a rapid recovery.—*American Medical Journal.*

**Arsenic in Lymphadenoma.**—In the *British Med. Journal*, Dr. Stephen Monckton records the case of a man, aged 37, who suffered from enlarged lymphadenomatous glands in the armpits and groins. The author decided to try arsenic in a new form of pill-preparation carried out at Hamburg, the principle being to invest the drug in keratin or horn-gelatine, in such a way as to render the pill insoluble in the acid fluids of the stomach, while it becomes readily dissolved in the alkaline contents of the upper bowel. A supply of pills was obtained from Bell & Co., each pill containing one-thirteenth of a grain of arsenious acid. The pills were commenced on April 5, and were continued until June 4, at the rate of three a day. During this time the glands everywhere gradually diminished in size; but, unfortunately, just at this time the patient was seized with pleuro-pneumonia and died. The author remarks that he had never seen glands disappear so rapidly under any other treatment.—*Quarterly Compendium.*

**The Treatment of Painful Fissure of the Anus Without Operation.**—The two plans of treatment generally recommended as the only reliable ones for the treatment of anal fissure are incision or stretching of the sphincter muscle. As a rule, perhaps, stretching may be preferred, as it causes no external wound, and therefore does not render the patient liable to septic poisoning. Mr. A. D. Macgregor (*British Medical Journal*, Feb. 27, 1886) treats such fissures without any operative interference at all, and he claims that his success is such as to warrant a continuance of his method. His plan is first to order a full dose of castor oil with some rhubarb; when this has operated the bowel is to be washed out with an enema of Condy's fluid. After passing the speculum, the fissure is to be then painted with a solution of chloride of zinc, 20 grains to the ounce, and a piece of lint smeared with boric ointment then introduced, the contraction of the sphincter serving to keep the lint in contact with the sore. The bowels are kept closed by opium, and liquid food only allowed. The subsequent treatment consists in the use of powdered boric acid half a drachm and violet powder 1 ounce, sprinkled freely on lint, and introduced into the anus to dry up any discharge. Mr. Macgregor states that one application of chloride of zinc in his experience has always proved sufficient, and only causes some smarting and uneasiness.—*Therapeutic Gazette.*

**Tobacco.**—From the *Lancet*, January 30, we learn that the trustees of the Fiske Fund at their last annual meeting announced that they had awarded the prize to an essay on the "Physiological and Pathological Effects of the Use of Tobacco" bearing the motto "Quid nimium probat, nihil probat." The author was found to be Dr. H. A. Hare, of Philadelphia. The conclusions at which the author has arrived are thus summed up:

Tobacco smoking does not decrease the urine eliminated, but rather increases it; tobacco does retard tissue waste; tobacco and its alkaloid cause convulsions in the primary stage of the poisoning by depressing the reflex inhibitory centres in the cord; it causes the palsy of the second stage by paralyzing first the motor nerve trunks and then the motor tract of the spinal cord; the sensory nerves are not affected by the drug; nicotine contracts the pupil by stimulating the oculo-motor and paralyzing the sympathetic, this action being peripheral; nicotine first lowers the blood-pressure and pulse-rate, secondly increases pressure and rate, and finally decreases pressure. The preliminary lowering of pressure and rate is due to pneumogastric stimulation, associated with vaso motor dilatation; the second stage is the result of vaso-motor construction and pneumogastric paralysis; the third is due to vaso-motor dilatation returning. Death by poisoning from this drug results from failure of respiration, the action being on the central nervous system. The blood-corpuscles are broken up and cremated by the action of the poison. In death from nicotine poisoning the blow shows changes in the spectra. Death can be brought about by cutaneous absorption of nicotine. Tobacco increases intestinal peristalsis in moderate doses, and produces tetanoid intestinal spasms in poisonous ones. The liver appears to destroy the poison, although this destruction is participated in by every set of capillaries in other parts of the body. Tobacco smoking increases pulse-rate and decreases arterial pressure.—*Ib.*

**Cocaine in Pruritus Ani.**—Mr. Malcolm Morris, in a note on "Hydrochlorate of Cocaine in Pruritus Ani," relates the case of a gentleman who had long suffered from this distressing complaint. A solution containing twenty-five per cent. of the drug, with five per cent. glycerin, was ordered to be painted over the extruded mucous membrane and neighborhood of the anus, three times at intervals of ten minutes, the parts being allowed to dry somewhat before moving after the third application. As the result, the patient slept quietly for seven hours. This method was persevered in night and morning for more than a week without any return of the pruritus; it was then omitted for two days, and the irritation returned as bad as ever, while resumption of treatment again gave relief. Dr. Cottle has tried the remedy in the following two cases:

1. A lady with extensive lichen planus and severe irritation, preventing sleep without narcotics; all usual local remedies were without benefit. A four per cent. solution of hydrochlorate of cocaine was freely and repeatedly applied to and around the spots without relief.

2. A lady with severe eczema of the limbs of long standing, the parts being red, exuding, and partially excoriated; there was most intense itching unalleviated by ordinary measures; a five per cent. ointment of cocaine in vaseline was freely and frequently applied, and rubbed in as firmly as tenderness of skin permitted; slight diminution of the irritation followed. He thinks if it is to do good it should be dissolved in fat or oil, and the condition of parts should be such as to allow of firm rubbing in so far as to favor absorption.—*Quarterly Compendium.*

**A Remedy for Hay Fever.**—Dr. A. F. Samuels, of Prairie du Chien, Wisconsin, writes to us that for years he had been tormented every summer with an aggravating form of hay fever, for which he had tried in vain every remedy that was brought to his notice, but that he finally hit upon an ointment made after the following formula:

| | | |
|---|---|---|
| ℞. | Powdered camphor, Chloroform, } aa | 1 drachm, |
| | Extract of belladonna | 4 grains, |
| | Bicarbonate of sodium | 20 grains, |
| | Benzoinated lard | 1 ounce. |

The camphor, chloroform, and extract of belladonna are first rubbed up, the benzoinated lard is then added, and finally the bicarbonate of sodium. The ointment is applied freely within the nostrils with the little finger. It gives him steady and permanent relief.—*N. Y. Medical Journal.*

**The Arsenic Habit—A Remarkable Case.**—Dr. T. D. Crothers reports, in the *Quarterly Journal of Inebriety* for Oct., a remarkable case of the arsenic habit. The patient, an English horse-jockey, had led an irregular life, and had probably drank considerably. He began to take arsenic about the year 1875, using it for the purpose of keeping off fevers. For five years he took from 1 to 2 drachms of Fowler's solution daily, and his general health continued good. From this time he gradually increased the dose until he was taking from 12 to 20 grs. of arsenic daily (!), either in the form of the powdered acid or of Fowler's solution. When seen in 1885, his appearance was that of good health, the face full, the skin clear and white, the eyes brilliant but unsteady, cutaneous sensibility was diminished, the appetite was poor, the action of the bowels irregular, and the heart was enlarged. There was no muscular weakness, but the gait was slow and hesitating.

THE *Kansas City Medical Record* holds that seventy-five per cent. of society discussions is rubbish, and that medical journals cannot publish them in full and live, and that no society can long force upon the public, by "Transactions" or "organs" material not worth publishing. We are much pleased to note this expression from such a source. Would that the secretaries of all the societies whose proceedings we have "respectfully declined," might frame it and hang it up conspicuously before the members at each meeting.

**Salicylic Lemonade.**—The *British and Colonial Druggist* says that as a "hospital beverage," which has lately been found of very great value in cases of typhoid and other fevers, scurvy and gout, the following cannot be too widely known; it having been, we understand, fiist devised by a late medical officer attached to the anexhibition:

℞. Fruct. limon.................................No. 10,

Acid citric..................................℥ ss,

Acid salicylic...............................grs. 200,

Sacch. alb., aqua, aa......................... q. s.

Squeeze the lemon and put the juice aside; boil the fruit in half or three-quarters of a gallon of water for fifteen or twenty minutes; atter standing six hours take out the lemons, and again press them before throwing the exhausted pieces away. Add the juice and citric acid to the liquid, boil five minutes, and strain. Whilst hot add the salicylic acid, and stir until dissolved. Sweeten to taste with the white sugar, and make up the bulk to one gallon with water.

Salicylic lemonade may be taken freely, either of the strength here given, or diluted with half its bulk of water. It should be freshly made every two or three days, unless it be permissible to "qualify" it by the addition of a little pure French brandy. If required to be in "bright" condition, add when cold, a little beaten up white of egg, boil for three minutes, and filter. If found rather too harsh for some tastes, dissolve in the boiling liquid, before straining, half an ounce of Nelson's patent opaque gelatine, previously swelled for five hours in cold water.—*Technics.*

**Rapid Cure of Suppuration of Middle Ear of Thirty-five Years' Standing.**—The *Medical Press* tells us that Dr. G. B. F. Brunetti, of Venice, reports the following:

The patient, a physician, æt. 40, had suffered from offensive ottorrhœa since his fifth year. Hearing power had gradually sunk to a minimum. The watch could only be heard on contact. The tynpanum was absent on both sides. The ossicles were present on both sides; on the left they were incompletely ankylosed. After cleansing the auditory passage and middle ear, iodoform and sp. vin. rect. were employed, and in two days the stench disappeared; after eight days a few drops only of pus escaped. For eleven days a 0.5 per cent. solution zinci sulph. was used, and then the iodoform and alcohol again. In one month the patient was discharged, the vegetations in the tympanic cavity which were at first present had disappeared; its coverings had become healthy looking. Whispering could be heard at one metre distance, and the watch at 65-200 (left) and 30-200 (right) of normal.

**Pilocarpin Injections.**—Dr. Humbert Molliere has successfully treated double pneumonia with pilocarpin; the patient, exhausted by dysentery and albuminuria, was attacked with pneumonia in both lungs, and the intestinal disturbance was greatly aggravated.

A centigramme (about 1-7 of a grain) of pilocarpin was injected; the respiratory movements fell from 48 a minute to 24, and dyspnœa was much relieved; four hours later, a fresh injection was made, and was repeated next morning; each injection was followed by profuse sweats and salivation, and dyspnœa was greatly relieved. The patient rapidly recovered. In administering pilocarpin, M. Molliere was guided by former experience. An elderly man with uremia, accompanied with dyspnœa and delirium, who seemed dying, was greatly relieved, and ultimately cured by a similar treatment. Dr. Molliere describes another case. A young woman with a comatose form of uremia and renal lesion, following a cardiac affection, was freed from the comatose condition by injections of pilocarpin.—*British Medical Journal.*

**A Portuguese Method of Treating Ringworm.**—Ringworm of the most obstinate character may, according to Dr. Saerlis, writing in the *Medicina Contemporanea* of Lisbon, be cured in ten days by cutting the hair from the affected spot, pouring turpentine on it, letting it run over the whole head, and rubbing well with the finger. After this has caused a smarting sensation from three to five minutes, it is washed off with carbolated soap. Hot water is then used for washing the whole head, and the affected spots touched with dilute tincture of iodine or with a 2 per cent. solution of iodine and turpentine. This process is to be repeated once or twice a day.—*Quarterly Compendium.*

**Treatment of Angina Pectoris.**—M. Huchard discusses this question in the *Union Medicale*, 134 and 136, 1885. After rejecting all medicines that narrow blood vessels or otherwise increase blood pressure, he alludes to nitrite of amyl, morphine, and glonoin as palliatives, but asserts that they are of no permanent benefit. Treatment by iodine, however, is really curative if continued for from fifteen to eighteen months in daily doses of 15 to 45 grains. He also recommends ignipuncture and blisters over the precordial region, and regulation of the diet and mode of life, including a partial or exclusive milk diet, forbidding all exciting and alcoholic substances, as well as tobacco. In advanced atherome a cure is not to be expected, but amelioration is frequently possible.—*Ibid.*

**Benzoate of Cocaine.**—Senor Alfredo Bignon, in a paper read before the Lima Academy of medicine, and published in *La Cronica Medica*, strongly recommends the employment of the benzoate of cocaine in preference to the hydrochlorate (the salt most commonly used), and to the salicylate and borate, with which he has also made experiments. He finds that the benzoate is extremely soluble, easily crystalizable, and retains the characteristic odor of cocoa itself. The antiseptic qualities of benzoic acid also are an additional advantage. Amongst other experiments, the anæsthetic effects of a 20 per cent. solution of the benzoate were compared with those of a similar solution of the hydrochlorate in a case of epithelioma of the tongue, with the result that the effect of the former salt persisted for a much longer time than that of the latter.—*Ibid.*

**The Digestion of Milk.**—Dr. M. Reichmann draws the following conclusions from a number of elaborate experiments as to the digestibility of milk in the human stomach (*Deutsche Medical Zeitung*, No. 82, 1885):

1. Boiled milk leaves the healthy stomach more rapidly than an equal quantity of unboiled milk.
2. The digestion of boiled milk is more rapidly accomplished than that of unboiled milk.
3. The coagulation of unboiled milk in the stomach is complete in five minutes.
4. This coagulation is not caused by the acid of the gastric juice, but by the influence of a special ferment (milk-curdling ferment).
5. The acidity of the gastric juice is at first due almost solely to lactic acid, and later in the process of digestion, to the presence of hydrochloric acid.
6. Hydrochloric acid first appears in perceptible amount forty-five minutes after the ingestion of half a pint of milk.
7. For the first hour and a quarter after the ingestion of milk the acidity gradually increases, and then decreases, until the milk has entirely left the stomach.
8. The curds of casein in digestion of boiled milk are much softer than in the digestion of uncooked milk.—*Therap. Gazette.*

**In Coryza.**—One of the best prescriptions to allay the irritation and sneezing and check the discharge, is composed of the following ingredients:

℞. Morphiæ sulphatis............................grs. 3,

Pulveris acaciæ..................................dr. ½,

Bismuth subcarbonatis.........................dr. 1.

M.

After cleansing the parts with Dobell's or some similar solution, this powder can be snuffed up the nostrils. However, it can be used without any previous cleansing of the parts, and will be found to give almost immediate relief from the above symptoms.

An attack of acute coryza may often be aborted by giving from six to ten grains of sulphate of quinæ, together with hot pediluvia and a hot lemonade just before retiring for the night. The object is to stimulate the sudoriferous glands and restore the proper harmony between heat-production and heat waste. A full dose of Dover's powder, (ten grains to an adult), will relieve pain, if any exists, and produce diaphoresis. Other remedies that sometimes used for the same purpose, are, carbonate of ammonia, ten to twenty grains; chloride of ammonia, thirty grains; solution of the acetate of ammonia, one ounce; tincture of belladonna, twenty minims. A very good way to relieve the unpleasant, "stuffed up" sensation in the nose is to take a drachm of pulverized gum camphor, put it in a quart of boiling water and inhale the steam through the nostrils.—*Lancet and Clinic.*

**Injections of Oil of Turpentine for the Radical Cure of Fistulæ.**—Cecchini has employed oil of turpentine with good results in the treatment of several varieties of fistulæ. He claims

**Chloride of Calcium in Glandular Enlargements.**—*Practitioner.* Arthur Davies, B.A., M.D., M.R.C.P. The author thinks its uselfulness is far from being generally known in this class of cases. He cites the particulars of a remarkable case under the care of his father, the late Dr. Herbert Davies. A gentleman, aged 37, had enlargement of the cervical, inguinal and axillary glands since childhood, gradually increasing in extent and hardness, those in his right axilla requiring him to sleep with his arm over his head. All treatment had been in vain. He was ordered 10 grs. of calcium chloride, three times daily, in 30 drops of water. In a month some diminution was observed. The dose was increased to 20 grs. On one occasion, *by mistake*, he took 35 grs. at a dose, and finding no unpleasant symptoms, increased the dose to *40 grs.*, *three times daily*, which he took for the greater part of the time during one year, without a single unpleasant symptom. The swelling of the cervical glands was almost gone, and the others very greatly diminished. This may fairly be called a case of lymphadenoma. The author has been very successful with it in the indolent enlarged lymphatics, in childhood. The dose should be gradually, yet cautiously, increased. Sometimes months elapse before benefit is derived—drug must be persevered in. It is of no use in suppuration.—*St. Louis Medical Journal.*

**Atropia Hypodermically to Prevent the Heart Depressant Effects of Chloroform.**—This drug is physiologically antagonistic to chloroform, it paralyzes the termination of the pneumogastric nerve in both heart and lungs—as proven by Drs. Ringer, H. C. Wood and others—and stimulates the sympathetic, which is exactly the reverse action of chloroform. I therefore concluded to try the hypodermic injection of one-twentieth of a grain of atropia at the next opportunity and watch the result. The subject was a lady, having already a weak heart and in a run down condition from the effects of a malignant disease of the breast (the skin being considerably involved). I gave her the above dose of atropia hypodermically, and then began the administration of the anæsthetic She took it without a murmur ; there was no trouble in breathing, no blue lips or nose, and the heart's beat was regular and even stronger than usual, and I completed the operation without a single bad symptom.

This was the first encouragement, and I concluded to try it in every case where an anæsthetic was needed, which I did, with similar results.

Care must be exercised, however, against too large doses of belladonna, lest the sympathetic nerve force become exhausted by over-stimulation.—*Dr. J. C. Kerr, in So. Cal. Practitioner.*

**Lactation and Medicaments** (Fehling, *Bull. de Therap.*, 30th August, 1885).—If two grams (half a dram) of salicylate of soda are administered, this substance is readily found in the urine of the new-born. The passage is especially marked when the drug has been absorbed two hours before the nursing. Iodide of potassium acts like the salicylate of soda. Iodoform, even when used in very small quantity, passes into the milk. A simple sprinkling of this drug upon the vulva is sufficient to secure its appearance in the mammary secretion. It was not so with corrosive sublimate, of which it was possible to discover in the milk only very small quantities—so small that it was impossible to estimate them. The narcotics are without effect upon the nursling. *The strongest doses of opium or of chloral administered to the nurses have not produced any special physiological effect upon the nursling.* Atropine tested upon animals produced dilatation of the pupil in the nursling only when the maximum therapeutic dose was exceeded.—*Edinburgh Medical Journal.*

**Corning on Local Anæsthesia.**—The author gives a brief summary of what others have done in the production of local anæsthesia by means of the salts of cocaine, and then he adds the results of his own researches. These show that simple arrest of the circulation in the part shortly after the injection of a solution of cocaine suffices to prolong and intensify the anæsthesia

Farther, if the injection be made after exsanguination and compression there is but little diffusion of the anæsthetic, and hence a commensurate diminution in the number of nerve filaments exposed to the influence of the solution. By the aid of massage some purely mechanical diffusion may be produced.

Again, if the injection is made a few moments before exsanguination and the application of the tourniquet, a sufficient amount of saturation of the tissue is obtained to expose a large number of nerve filaments to the influence of the anæsthetic.

By this method anæsthesia can be prolonged for any length of time, with comparative small quantities of cocaine.

It is essential in the use of this method to retard or stop the circulation of the affected part during the period required for the anæsthesia. In different portions of the body different forms of compressors are called for. The anæsthetic influence can be made to extend as deep as may be desired. The practical results of this method give great promise that a vast number of operations may be rendered painless without the resort to general anæsthesia.

The work is worthy the careful study of every practical surgeon and physician. It is clearly written, with little useless padding. The author stops when he has said what he wishes.—*American Lancet.*

**Suicides.**—The London correspondent of *American Practitioner and News* in a report of an interesting lecture by Prof. Quain writes that the amount of suicides varies with the seasons, forming a regular annual curve, of which the minimum is in December and the maximum in June.

The commonest method of suicide is hanging, then follows in order, drowning, cut, or stab, poison, gunshot. Women, however, select drowning before hanging, and poison before cut or stab. Women also differ from men in the selection of poisons, men choosing painless and sure preparations, while women take any poison that is at hand, indifferently. The choice of method is also affected by age, the young showing a comparative preference for drowning, poison, and gunshot; and by occupation, men using preferably the instruments of their crafts; and by reason, drowning avoided in the cold months. At the conclusion of the reading of the paper a discussion followed. Dr. Longshaff argued that education had a distinct tendency to increase the suicide-rate. Mr. R. Giffen said it appears to him a very remarkable fact that there was an increase of suicide in the summer months, and he should have liked more information on that head. Dr. R. Lawson, Inspector General of Hospitals, explained the extraordinary death-rate from suicide by soldiers in the army by the circumstance that the army was a refuge for those to whom all other resources had failed.

that this subtance acts as a powerful antiseptic, produces granulation, and can never do harm if a reasonable amount of care is exercised.

1. *Fistulæ in Ano.*—Seven cases treated, with five cures. The turpentine was injected by means of a syringe. As it causes considerable pain, it may be diluted with almond or olive oil. Short fistulæ are most easily cured by this remedy.

2. *Caries of petrous portion of temporal bone.*—Four cases cured in from two to three months. Boracic acid was used in conjunction with the turpentine. The discharge of fetid pus soon ceased, and complete cure rapidly followed.

3. *Dental fistulæ.*—Eight fistulæ, with caries of alveolar process and maxillaries, were completely cured.

4. *Fistulæ of Steno's duct.*—The fistulæ treated was caused by an abscess following a gun-shot wound. From the parotid region, fistulæ extended into the cheek, angle of jaw and neck. During mastication saliva flowed through the opening in the cheek. The fistulæ was healed in thirty days, with six injections.

Cecchini has also employed turpentine in the treatment of carbuncles and in post-mortem wounds, in every case with excellent results.—*Centralblatt for Chirurgie.*

**Cimicifuga for Headache.**—The severe headache which frequently ushers in the menstrual period is relieved by cimicifuga. Congestive, nervous and rheumatic headache, and those resulting from alcoholic excesses and loss of sleep, also frequently yielded to this drug. It is best given in small doses, say m. x of the fluid extract, frequently repeated. It has been found useful in the chorea of children.—*Texas Courier-Record.*

**To Arrest Nasal Hemorrhage.**—We take the following practical suggestion of Prof. John Cheiene, from the Edinburgh Medical Journal: In persistent hemorrhage from the nasal cavity, plugging the posterior nares should not be done until an attempt has been made to check the hemorrhage by firmly grasping the nose with the finger and thumb, so as completely to prevent any air from passing through the cavity in the act of breathing. This simple means, if persistently tried, will in many cases arrest the bleeding. The hemorrhage persists because the clot which forms at the rupture in the blood-vessel is displaced by the air being drawn forcibly through the cavity in the attempt of the patient to clear the nostrils. If the air is prevented from passing through the cavity, the clot consolidates in position and the hemorrhage is checked.—*American Practitioner.*

**How to Treat Bubo.**—This is a question often put to us. Opening the bubo? No. We have long ago discovered, and have taught our class for several years, to draw off the pus with an aspirator, and in the absence of one, to use the ordinary hypodermic syringe for this purpose in this way. After the pus is removed we inject an ethereal solution of iodoform into the abscess; the ether is absorbed and the iodoform is very evenly deposited on the walls of the abscess. Then we paint the bubo with collodion in which iodoform is dissolved; apply several coats. These, as they dry contract and compress the bubo, so we have the benefit of pressure in addition to that of the iodoform stimulant and antiseptic action inside. Under this treatment the sores on the penis heal more regularly and rapidly. This practice is better than the slashing one.—*Medical Advocate.*

**Artificial Feeding of Infants.**—Dr. Arthur V. Meigs, of Philadelphia, (*Phil. Co Med. Soc.*) recommends the following for young infants who must be hand fed: Obtain packages of milk-sugar containing 17¾ drachms each. The contents of one of these packages is to be dissolved in a pint of water, and when the infant is to be fed, there must be mixed together two tablespoonfuls of cream, one of milk, two of lime-water, and three of sugar-water, and this, when warmed, is ready for use The milk and cream to be used, it is hardly necessary to say, should be of the average sort; neither very poor nor adulterated, nor, on the other hand, should the rich milk or cream of fancy cattle be used, as in that case the proportions would have to be modified.—*Epitome.*

**Cocaine in Tonsillotomy.**—Marcel Leromoyez, in the *Bulletin de Therapeutique*, discussing the subject of local anæsthesia in tonsillotomy, recommends the hydrochlorate of cocaine. This he had used with good success in a solution of one in thirty. He painted the tonsil four times, five minutes elapsing between each time. Five minutes after the last painting, the tonsil was so anæsthetized that one could stick a knife three centimetres deep into it. The action continued ten minutes after the operation, and then a burning sensation commenced.—*Texas Courier-Record.*

THE St. Louis *Medical and Surgical Journal* gives an account of the removal of a brass ring from the penis of a boy, after amputation had been suggested by an eminent surgeon as the only remedy, by immersing the member in a bowl of metallic mercury. In a few minutes the ring which had been so imbedded in the tissue as to become invisible, crumbled out in pieces of amalgamated brass from the sadly disfigured organ. The hint is an exceedingly valuable one.—*Medical Age.*

